# An embodiment of the cinematographer: emotional and perceptual responses to different camera movement techniques

**DOI:** 10.3389/fnins.2023.1160843

**Published:** 2023-07-03

**Authors:** Mehmet Burak Yilmaz, Elen Lotman, Andres Karjus, Pia Tikka

**Affiliations:** ^1^Baltic Film, Media, Arts and Communication School, Tallinn University, Tallinn, Estonia; ^2^School of Humanities, Tallinn University, Tallinn, Estonia

**Keywords:** camera movement, embodied cognition, embodied simulation, cinematography, cinematographer, dolly, Steadicam, handheld

## Abstract

We investigate the relationship between camera movement techniques and cognitive responses in audiences, reporting on an experiment exploring the effects of different camera movement methods on viewers' degree of immersion and emotional response. This follows directly from preceding experimental literature and is further motivated by accounts and experiences of practicing cinematographers (authors included), which indicates a correspondence between the two. We designed three different cinematic scenes with indifferent moods, and shot each one time with Steadicam, dolly, handheld, and static camera, resulting in 12 different clips. A total of 44 non-professional participants watched the clips and rated their reactions in terms of arousal and degree of involvement. Experimental results are mixed: movement affects the sense of involvement but not necessarily emotional response. We present and discuss some further explorative results and possible future directions to improve the design. We argue in this contribution that there is value in experimental approaches to cinematography, enabling the systematic study of creative intuitions and audience responses in controlled settings.

## 1. Introduction

The cognitive and emotional significance of cinematography is well acknowledged in film studies. However, different techniques of camera movements (e.g., dolly, Steadicam, and handheld) in narrative storytelling are surprisingly sparsely studied topics. Intuitively, audience experiences may vary depending on *how* the camera is moved, and what type of viewpoint it generates for the viewers. Drawing from the tacit knowledge accumulated by cinematographers (including three of the authors), we argue that when producing specific movements, cinematographers may be viewed as *extending* their perceptual bodily senses *via* the camera onto the screen, and in this sense, these movements may be further seen to become *embodied* in the viewer's experience.

Throughout the article, the term *immersion* refers to the viewer's intense mental involvement in the filmic world. In other words, it represents the degree of the feeling of “being there”. *Involvement*, in return, is the perceptual and sensational (e.g., valence/arousal) association with onscreen events. *Engagement* describes the act of involvement or immersion itself. We utilize the concept of *embodiment* from Varela et al.'s (1991) perspective which highlights the vital role of the body with its sensorimotor capabilities in the process of cognition. Finally, *experience*, in our case, is an umbrella notion that encompasses the aforementioned terms and can be defined as the totality of one's sensory and motor reactions during movie-watching.

Our study has three objectives. First, to explore how different camera movement techniques impact the audience's emotional engagement with onscreen events in three varying mood situations. We explicitly test the hypothesis that a moving camera elicits stronger responses than a static camera and explore and visualize the outcomes of all the combinations. More broadly, we aim to understand how these techniques influence the degree of immersion (embodiment) of the viewer, and to what extent can theories, such as embodiment, be tested using experimental paradigms. Finally, we also seek to explore improvements in preceding experimental literature by creating more naturalistic, filmic stimuli and discuss their implications. We begin by introducing the theoretical background and cinematographic accounts that motivated the study.

### 1.1. Theories of embodied camera

It is widely acknowledged that the stature of camera movements is one of the key elements in film production that conveys the story, creates moods of the scenes, and emphasizes the internal struggles of the characters. Revealing the lifelikeness of events on the screen and absorbing the audience into the fictional reality, the moving camera is one of the most potent cinematographic tools that reduce the gap between viewers and the mediated world of fiction (Morgan, 2016).

The first moviegoers in the early 20th century enjoyed the commonly called phantom rides, the effect provided by the moving camera as it traveled on the train tracks and the canals of Venice as if being moved by an invisible phantom (Salt, 2009). By the 1930s, filmmakers explored the possibilities of moving the camera to an extent that a 1932 issue of *American Cinematographer* notes a meeting between directors and cinematographers over redundant execution and the abuse of camera movements (Hall, 1932). In 1930s Hollywood, the moving camera of the cinematographer Karl Freund (American Society of Cinematographers [ASC]) in *The Last Laugh* (dir. F. W. Murnau) “opens the film by descending in an elevator and gliding across a hotel lobby”, the scene that Luci Marzola acknowledges in the *Journal of American Cinematographers* as “perhaps the single most talked-about camera technique in the history of motion pictures to that point” (Marzola, 2020, see also in Müller, 2014; Keating, 2019). Another European émigré to Hollywood, Serbian filmmaker Slavko Vorkapich argues that similar to the innate human appreciation of motion, which is already present in newborn babies, adult film viewers must be drawn to and feel “pleasure” by motion suggested by the moving images (Vorkapich, 1930, p. 30). He also linked the perception of motion on the screen directly to the bodily sensory-motor experiences of the spectators: “By merely seeing a motion on the screen, our minds, conscious or subconscious, may be made to react in a similar manner as in active participation” (Vorkapich, 1930, p. 30). Half a century later, Vivian Sobchack appraised the moving camera as perceived “as always meaningfully-directed, as *intentional*: the unifying embodied activity of a human consciousness as it is situated in and inhabits the world” (Sobchack, 1982, p. 317). Christian Metz wrote “because movement is never material but is always visual, to reproduce its appearance is to duplicate its reality” (Metz, 1991, p. 9).

These arguments bear compelling resemblances to the cognitive theories of *embodied mind* (Varela et al.'s, 1991), *embodied simulation* (ES; Gallese, 2007), and their application to the field of cinema (Tikka, 2008; Gallese and Guerra, 2012; Tikka and Kaipainen, 2014). Several functional neuroimaging studies have shown that different film viewers' brain activations may correlate in a time-locked manner when they watch the same films (see, Jääskeläinen et al., 2020, 2021, for reviews). In this regard, cinema has the ability to convert the act of seeing that each individual experiences idiosyncratically into an “intersubjectively extroverted” vision (Sobchack, 2016, p. 64). Many of these studies also provide evidence that the observed events on the screen are simulated, or mirrored, in the viewer's mind (Gallese and Guerra, 2014). According to Sobchack, “cinematic ‘language' is grounded in the language of embodied existence” (Sobchack, 1992, p. 13), and the “moving image is not only perceptible, it is also perceptive” (Sobchack, 2016, p. 75).

Recent neurocinematics research on human mental projections of cinematic techniques has proliferated and produced promising results (Zacks, 2010; Heimann et al., 2014, 2019; Kauttonen et al., 2014, 2018; Raz and Hendler, 2014; Kovács et al., 2019). However, to the best of our knowledge, there are only a few cognitive or neuroscientific studies about the effects of camera movements in the context of embodied cognition. Heimann et al. used electroencephalography (EEG) to measure the neural motor responses of viewers who watched camera movements produced by Zoom, dolly, Steadicam, and stationary cameras (Heimann et al., 2014, 2019). Results demonstrated that Steadicam stimulated the corresponding areas of the brain more than the other techniques, meaning that it afforded the strongest bodily engagement. This supports the view that “film's intentionality and subjectivity are also grounded on the viewers' embodied simulation of camera movements, suggesting that the immanence of cinematic subjectivity largely relies on the bodily nature and understanding of film” (Guerra, 2015, p. 153). These first studies bear significance as they produced experimental evidence supporting the argument that different techniques of camera movements alter the degree of embodiment. Guerra suggests following up with “an ES-based approach, deepened with specific experiments on different stylistic solutions, could also explain stylistic changes caused by the constant evolution of viewers' ability to play a role in a virtual world” (Guerra, 2015, p. 151).

However, from our point of view as filmmakers, the video stimuli presented to participants were not aesthetically appealing in terms of cinematographic imagery and lacked certain aspects usually present in fiction films, such as dramatic lighting and narrative qualities. Hence, it might be debatable to which extent the results could satisfactorily explain effects related to viewing camera movements in cinema. We aimed to improve on this ecological validity aspect in this study.

Cognitive–perceptual theories applied to film studies often emphasize the analogy between a moving camera and the human eye (e.g., Bordwell, 1977; Sobchack, 1982; Barker, 2009; Guerra, 2015; Schonig, 2017). The key arguments are 2-fold. First, the viewpoint that certain camera movement techniques (e.g., Steadicam and handheld) depictions of the world are arguably analogous to the dynamic movements of the human eye (Schonig, 2017). Second, such camera movement on the screen appears to be “the closest approximation of muscular movement of the human body” (Barker, 2009, p. 110). Hence, the perceptual implications of the moving camera on the projected images resemble those of the bodily movement of the humans in the actual space. Similar to our innately mobile bodies, moving camera's frames' “articulated and finite boundaries orient and organize the viewing view's perceptual and motor access to—and in—the film's world.” (Sobchack, 2016, p. 72). Therefore, “the frame's spatiotemporal coherence and relative constancy (even when the viewing view/viewed view within its bounds is moving) significantly synthesizes the viewing view's sense perception and movement into a particular and unified ‘place”' (Sobchack, 2016, p. 72–73).

In this sense, if considered from the cinematographer's point of view, the human eye in the viewfinder of a camera extends its cognitive–perceptual abilities to the camera (technology). When the camera starts moving, this bodily extension not only engages the visual system but also the whole body of the cinematographer. The camera may be said to become an embodied extension of the cinematographer's body and mind. This can be understood in line with the embodied mind views to the body–brain–world interplay, in general, and to human creation in arts, in particular (Vesey, 1965; Gibson, 1966; Lakoff and Johnson, 1999; Grodal, 2002; Tikka, 2008; Coëgnarts, 2017; Coëgnarts and Slugan, 2022). However, it is important to acknowledge that certain mobile frames, such as cranes, drones, and vehicle-mounted shots, do not precisely correspond to human biological and muscular movements. Such movements can be assumed to be exempt from the eye–camera analogy. On the other hand, our gaze or kinesthetic movement does not always have to perfectly match that of the camera to perceive and comprehend the movement. Whether conscious or subconscious, we can still identify the cinematic world and orient ourselves in it by viewing even inhuman movements according to or with the non-anthropocentric dynamics of alternative filmic reality (Sobchack, 2016, p. 86).

We propose that the creative cognition of cinematographers in the process of image-making, as well as that of the viewers, may embody, and psycho-physiologically simulate the movements of the camera as if they were moving themselves (Coëgnarts, 2017; Tikka, 2022). In Henderson's words, “cinema overcomes two-dimensionality through its 'walk-around' capability” (Henderson, 1970). Such a relationship between movement (either by mobile frame or other similar means) and space is so essential that without it, Sobchack claims, there would be no cinema (Sobchack, 2016, p. 66). Garrett Brown, the inventor of Steadicam, puts it simply: “We are there”—similar to a human eye, the moving camera explores moment-by-moment missing information related to the physical story space (Brown, 2003). For the viewer, camera movement provides an experience of “subjective movement through an objective world” (Bordwell, 1977, p. 23).

### 1.2. Camera movement from ecological perspective

According to the ecological view, exploration of one's surroundings by means of a coordinated interplay of vision and sensorimotor locomotion allows for detecting affordances for goal-directed actions in the natural environment (Gibson, 1966). The Gibsonian perception–action loop assumes the dominance of the visual system over the motor system. Contemporary views on human cognitive inference abilities to explore space assume continuous prediction coding in the brain for immediate updates of the optic information (Tivadar et al., 2021). Prediction errors describe the neural bottom-up processes involved when unexpected events instead of expected events occur in the optic field (Alefantis et al., 2022).

Gibson's ecological views on perception have been adopted and widely applied in the field of cognitive film studies (e.g., Detenber and Reeves, 1996; Anderson, 1998; Anderson and Anderson, 2005; Cutting, 2005; Smith, 2012; Tan, 2018). The neural prediction coding and prediction error may be assumed intrinsic to the affective–cognitive sense-making processes that take place when perceiving continuously unfolding narrative information. Indeed, prediction coding and error can be considered as a systemic counterpart in the brain for the viewer's perception of false cues and false information which at some point deliver surprise for the viewers. Camera movements play an important part in both hiding something from the viewer as well as directing the viewer's attention to that something.

In the context of watching a movie, it can be assumed that the flow of optical arrays (light) generated by the moving camera and then projected on the screen are to some extent perceptually similar to physically moving in space. If a visual system is dominant over the muscular, according to Gibson, then the viewers could easily surrender to the visual illusion that they are moving with the camera in the narrative space, although they are physically stationarily seated in the cinema chair. In a similar vein, Detenber and Reeves (1996), regarding the perceptual responses to the film, claim that “there is no switch in the brain that deactivates them just because the stimulus is mediated rather than real” (Detenber and Reeves, 1996, p. 78). The perception of the animated screen operates akin to the perception of real life, but viewers also learn how to comprehend film and television footage through repeated experiences (Salt, 2009, p. 32). Visual perception alone, with or without locomotion, does not enable the cognitive mapping of space. Constructing an understanding of perceived space further involves experience and memories of moving around in one's environment (Neisser, 1980).

A significant component of human engagement with the arts and sciences relies on the human ability to mentally simulate situations, actions, and the consequences of those actions without moving a muscle in their body. Cinematographers can imagine their camera movements in a given space based on the memory of the space, without any locomotion. Furthermore, the seated viewer is constrained in their bodily movements. Drawing from Bolens' (2012) account, Müller (2014) suggests that perceived bodily movement onscreen stimulates the sensorimotor system, and the viewer reconstructs the same locomotion mentally. In return, although partially illusory, the analogous movements on screen and viewers' perceptions allow viewers to establish a stronger emotional engagement with onscreen events (Bolens, 2012; Müller, 2014). In this sense, the motion may influence emotional responses (Simons et al., 1999).

### 1.3. Related studies in psychology

Mühlberger et al. (2008) assessed the emotional impacts of looming, receding, and static pictures on viewers in three different contexts: pleasant, unpleasant, and natural. The results demonstrated that the unpleasant looming pictures elicited strong responses both in valence and arousal ratings. The outcomes support the claim that alteration in physical distance affects emotional responses. Furthermore, the authors suggest that more sophisticated stimuli materials should be used for future experiments on the subject (Mühlberger et al., 2008). Several studies have evaluated the influence of motion stimuli on emotion (Detenber and Reeves, 1996; Detenber et al., 1998; Simons et al., 1999, 2000). Participants were shown still, and moving versions of the same clips were obtained from film and TV footage. Subsequently, their valence and arousal ratings were measured with self-reports and physiological measurement tools (e.g., skin conductance response and heart rate). Despite the different measurement tools and experiment designs (e.g., within-subjects vs. between-subjects) within the three experiments, the findings were consistent. Both in self-reports and physiological measurements, compared to still images, moving images appeared to be more arousing irrespective of whether the image was positive, negative, or neutral. The authors conclude that “the impact of image motion on the image-induced emotional response is inherent to motion itself” (Simons et al., 2000, p. 708).

In another study, Visch and Tan (2009) presented participants with different animated films in which moving objects depict chase scenes varied as five parameters—velocity, efficiency, fluency, detail, and deformation. Subjects categorized these clips into four genres (non-fiction, comedy, drama, and action) and also rated their emotional responses. Findings revealed that merely by watching different object movement patterns, viewers were consistently able to categorize them into genres. Furthermore, the movement patterns identified as fiction genres and their corresponding emotional reaction ratings were in line (e.g., comedy = response “funny”, drama = “sad”, and action = “impressive”) (Visch and Tan, 2009). Overall, the above-mentioned studies in psychology exhibit guiding results concerning the emotional repercussions of motion, which can be applied to the context of camera movements in cinema.

### 1.4. Different ways of moving the camera

When faced with choices concerning camera movements and storytelling, cinematographers intuitively turn to their tacit knowledge (Calhoun, 2003; Pavlus, 2003; Lotman, 2016, 2021). Due to the nature of their work, filmmakers are often concerned with practical and narrative questions, such as whether there is a motivation to move the camera or whether executing a certain camera movement at any particular point would help the story (Nielsen, 2007).

Our study focuses on three established moving camera techniques: dolly, Steadicam, and handheld, which are compared to static cameras. We are interested in whether their distinct qualities produce different experiences for the viewers. Dolly is a wheeled cart-like device with a mounted camera. It can either be put on track or simply used on its own wheels. Dolly creates a smooth and stable movement. Steadicam is a special camera stabilization system invented by camera operator Garrett Brown in 1975. The Steadicam operator wears a vest that has an artificial arm attached that absorbs any friction of camera movement. The camera is mounted on the Steadicam sled which is connected to the arm. An important part of the Steadicam stability is not only the skill of “flying” it but also the skill of balancing the rig depending on the shoot (against the gravity with the drop-down speed and against the centrifugal force). Unlike the bulky and heavy dolly, Steadicam is more flexible to use and allows its operator to roam around freely. Steadicam generates smooth and stable movement akin to the dolly. However, depending on the skill level of its operator, the sense of slight “human touch” could be perceivable. Finally, as the name suggests, a handheld camera is a technique in which the camera operators operate the camera placed in their hands, shoulders, or hips, depending on the size of the camera. In contrast to dolly and Steadicam movements, handheld cameras may generate unstable images. The choice of the type of camera movement is made depending on the style and genre of the film in production, and what type of emotional experience the images are designed to convey for the viewers.

The chosen camera movement and related technical devices each have their different implications on the nature of the movement and how the viewer perceives it. They cause altering effects on the emotional engagement and bodily involvement of the viewer in the given scene. Thus, understanding the affective functions of different camera movement techniques is crucial for filmmakers. For instance, director of photography Vittorio Storaro (ASC) argues that compared to the limitations of dolly, Steadicam allows him to convey the “rush of feelings between the main characters” and determine an emotional state (Ferrara, 2001, p. 147). Steadicam inventor Garret Brown suggests that “Steadicam shots most closely resemble what humans see through our remarkably-stabilized eyeballs as we navigate our own daily ‘movies”' (Pennington, 2020). Another Steadicam operator Ted Churchill states that Steadicam has the capability of “scaring half of the country to death” due to the sense of involvement it affords (Churchill, 1983, p. 119). In line with Brown, Jeff Mart also claims that unlike the sense of glide that dolly fabricates, Steadicam produces slightly wobbly and “less-than-perfect motions”, which is closer to the genuine human experience of moving (Comer, 1993, p. 78). Director John Carpenter characterizes handheld shots as “moving chaos” (Ferrara, 2001, p. 114). Such examples illustrate the filmmakers' perspective that there are fundamental differences between the ways the camera is moved.

### 1.5. Embodiment of the cinematographer

The theories and practitioners' accounts presented above stress the cognitive and narrative potential of camera movement. However, they often attribute such potencies to the camera device itself as an autonomous entity, while disregarding the creative and cognitive mind behind the moving camera, the cinematographer. In our view, the cinematographer is the main source of the embodiment simulation of the viewer, and the camera is a means at their disposal. In movies, “the body of the spectator, the body of the film, and the body of the filmmaker” are intertwined (Gallese and Guerra, 2012, p. 189). The body of the film is the reflection of the author's embodied knowledge which, in return, is simulated by the viewers. MacDougall (2006) explains “…any image we make carries the imprint of our bodies […] They are, in a sense, mirrors of our bodies, replicating the whole of the body's activity, with its physical movements […] Corporeal images are not just the images of other bodies; they are also images of the body behind the camera and its relations with the world” (MacDougall, 2006, p. 3). Hence, cinematographers and directors “create, layer by layer, a living object sharing perceptual and cognitive structures with its viewer” (Gallese and Guerra, 2012, p. 189). In this sense, this filmic world, constructed by what Gallese and Guerra (2012; 190) refers to as the “filmic cognition of filmmakers”, presents an “interaction between author's embodied knowledge gained in the real-life-situatedness and the modified representations of author's embodied knowledge gained therein” (Tikka, 2006, p. 146).

Tikka (2022) recently proposed a model labeled “Enactive Authorship”. According to this proposal, embodied experiences of the author are simulated by the viewer *via* the protagonist's situatedness, and the author is the “embodied cognizer” (Tikka, 2022). We consider the camera as an active participant in any given scene and as the eye of the spectator. We propose to extend this model and argue that in the case of camera movement, cinematographers take the role of embodied cognizers and utilize their embodied experience, which is deeply rooted in their tacit knowledge, to create the movement of the camera in their prefilmic mental space to manipulate the emotions and the perception of viewers in accordance with the needs of the story. In exchange, when executed in filmic physical space and appearing on the screen, the mobile frame triggers the embodied simulation of the viewers and links them with the deliberately manipulated version of the cinematographer's cognition. If “film style creates the condition for an embodied film cognition” as Guerra suggests (Guerra, 2015, p. 143), then we would argue that it is the cinematographer who sets up such conditions.

We do not suggest that cinematographers must be given all the credit for devising a camera movement. Naturally, directors are also involved in creative decisions. However, the creative input of cinematographers in films is often overlooked in academic texts. For instance, reflecting on Deleuze's critique of Alfred Hitchcock's *Notorious*, Gallese and Guerra (2012 p. 200) wrote “Hitchcock aims to contact the viewer at a pre-cognitive level exploiting the potentiality of camera movements, and promoting an embodied approach capable of enhancing the suspense effect” (Deleuze, 1986; Gallese and Guerra, 2012). This account is in line with our proposal, as it exemplifies the deliberate cognitive input of authors to simulate viewer embodiment. However, it disregards the contribution of cinematographer Ted Tetzlaff (ASC) and gives all credit to Hitchcock.

An insight motivating the experiment in this contribution is that cinematographers can be understood as “embodied cognizers,” who extend a modified and manipulated version of their bodily perceptions on the screen to viewers via camera movements. Cinematographers are likely intuitively aware of the different sensational and perceptual implications of different camera movement techniques through tacit knowledge and utilize this embodied understanding effectively. As testing the intuitions of cinematographers would be a challenging task, we start by exploring the effect of camera movements (and lack thereof) on viewers' emotional states in a controlled experimental setting.

## 2. Methods and materials

### 2.1. Stimuli

The stimulus scenes were designed to resemble scenes from actual movies in order to provide an experience more similar to watching a movie for the participants. Three scenes of different moods, suggesting an erotic encounter, a horror/thriller, and an ambiguous mood were each shot with four camera techniques, Steadicam, dolly, handheld, and static, resulting in 12 clips of approximately 45 s each. The zoom technique was not included as, in our view, zooming merely magnifies the image mechanically, instead of enhancing the embodied illusion of getting physically closer to an object in space. In this sense, zoom is “more transitive than transformative” (Sobchack, 2016, p. 80). Stationary shots were included to enable comparison with the moving ones.

In order to ensure that the main contrast would be the camera movements, we reduced other sources of variation while still ensuring the presence of essential visual elements of moody cinematic imagery. Visual components of the scene, such as lighting and placement of objects, were kept identical in all 12 stimulus variations. Furthermore, the use of music, sound, and human actors was avoided to keep the focus on the differences in the camera. While we made an effort to do so, in naturalistic stimuli like this, it is not possible to wholly eliminate the effects of other cinematographic variables such as focal length, shot size, lighting scheme, and framing. This is a tradeoff between ecological validity (naturalness) and experimental control.

**Figure 1:**
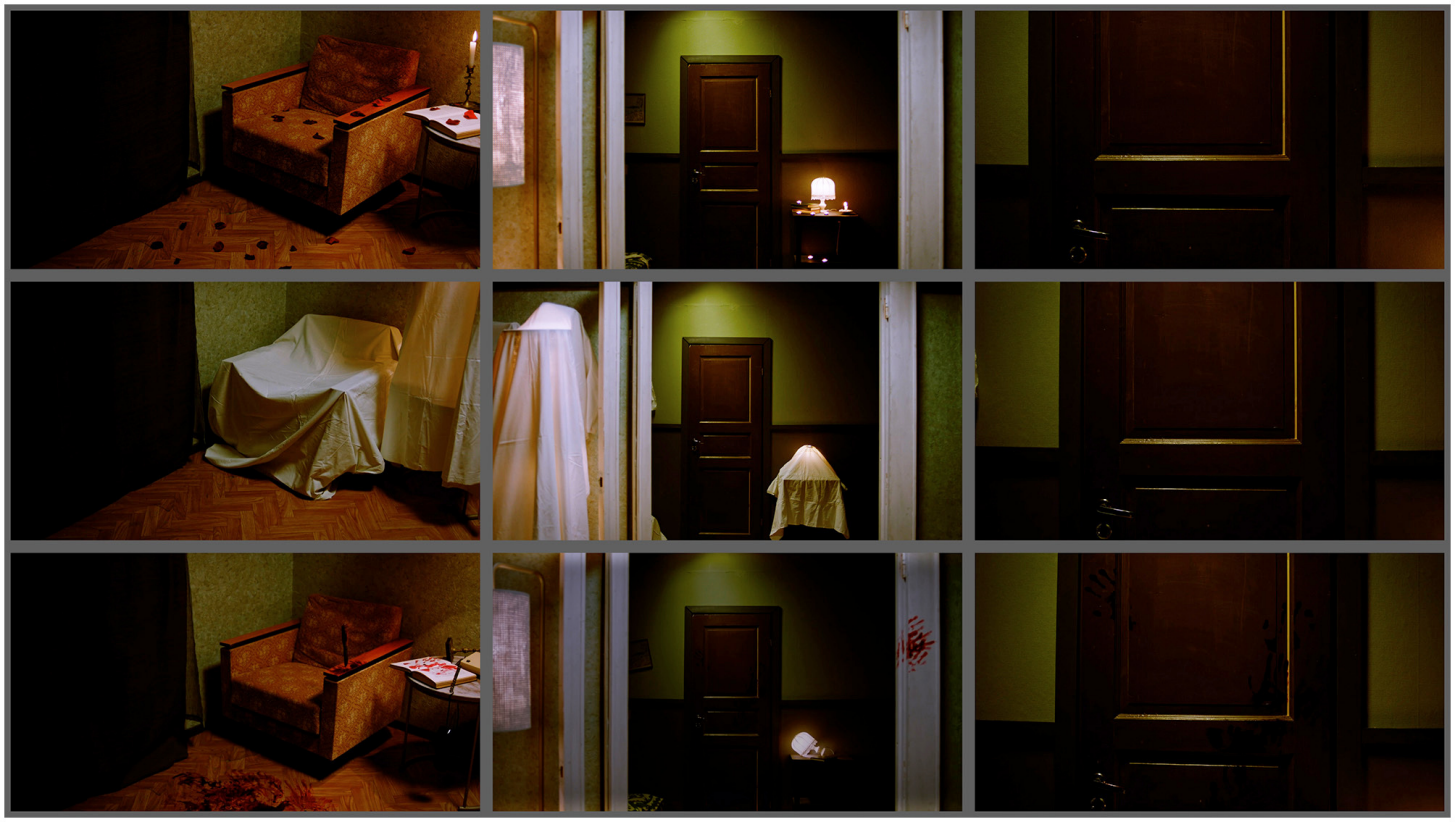
Top to bottom: frame grabs from pleasant, neutral, and unpleasant scenes.

**Figure 2:**
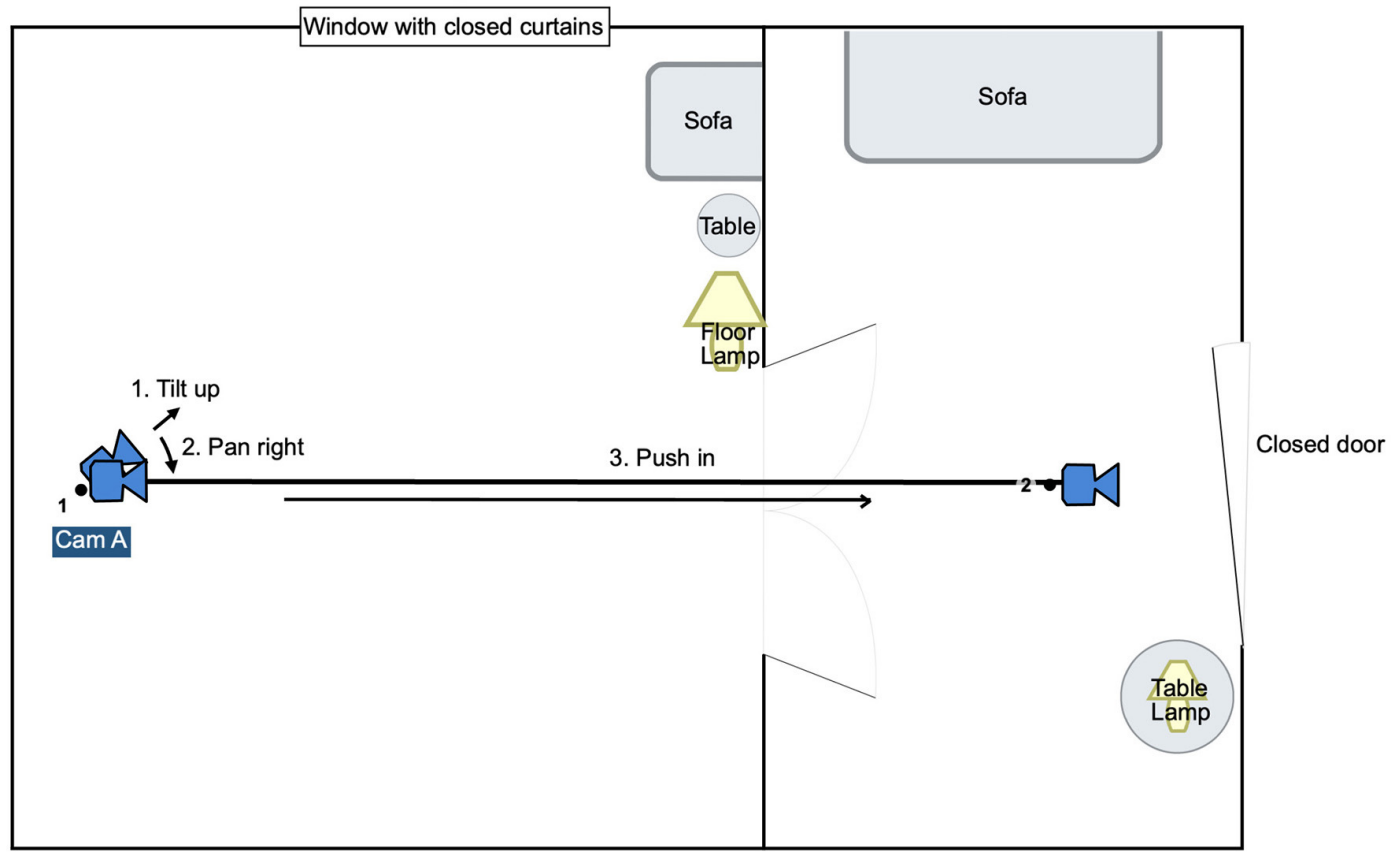
Floor plan and schematics of the stimuli scenes.

In all three mood scenes, the scene starts with the camera tilting up from the ground and panning to the right. Each scene contains specific visual clues, such as blood trails on the ground in the horror scene and red rose petals in the romantic encounter. After the up-tilting, the camera reaches eye level, it starts moving toward a door by means of either Steadicam, dolly, or handheld and stops right in front of a closed door. Figure 1 shows frame grabs from all three scenes and Figure 2 illustrates the movement path of the camera. The starting and the ending frame of the camera movement as well as the pace and the duration of the movement were kept similar. In the stationary or static camera condition, the camera stays stable after tilting up and panning right. In cinematic terms, the camera movement refers to the physical displacement of the camera. When the camera is fixed with a tripod or any other means, the shot is often considered static, even if it pans or tilts.

All mood scenes were produced with a team of film professionals using professional cinema equipment. A cinematic lighting scheme was created in a controlled studio environment. The footage was captured by Arri Amira in 2K ProRes 444XQ LogC format and color graded in the DaVinci suite to further match contemporary professional film production standards.

All the stimuli scenes are open access. Access links to the materials can be found under the data availability statement section at the end of the article.

### 2.2. Participants

In total, 28 healthy women and 16 men (between the age of 19 and 68, with an average age of 28) participated in the experiment. The participants were recruited through university e-mail lists and Facebook groups. All subjects gave their written consent to participate in the study after orally being informed about the procedure and the duration of the experiment. No risk factors listed in the Ethics Committee of Tallinn University guidelines were present during the experiment and the ethical principles of informed consent were followed. To compensate for their time, each subject received a gift card to a local bookstore after the experiment had been completed.

### 2.3. Experimental design and procedure

Out of 12 clips, four stimulus sets were combined, each of which consisted of three different mood clips and three different camera movements. The underlying thought was to present each participant with only one camera movement variation from each mood condition in order to avoid familiarization by repetition. Hence, with that being the only condition, 12 clips were shuffled into four groups randomly (see Table 1 for stimuli sets). As a result, each participant saw three clips and each clip was seen a total of 11 times.

**Table 1:** Stimuli sets.

	**Erotic (Positive)**	**Ambiguity (Neutral)**	**Horror (Negative)**
Static (STA)	1	2	3
Handheld (HH)	2	1	4
Dolly (DOL)	4	3	1
Steadicam (STE)	3	4	2

Set 1: Ambiguity (HH)—Erotic (STA)—Horror (DOL).

Set 2: Ambiguity (STA)—Erotic (HH)—Horror (STE).

Set 3: Ambiguity (DOL)—Erotic (STE)—Horror (STA).

Set 4: Erotic (DOL)—Ambiguity (STE)—Horror (HH).

As participants were asked to write their ratings on separate paper assessment forms, each session started with oral instructions about how to fill out the assessment form after each film clip. Each session started with the showing of two training clips extracted from two feature films, in order to familiarize them with the alternating process of viewing a clip and immediately filling out the assessment form.

The first clip (60 s) was a Steadicam shot from “Goodfellas” (dir. Martin Scorsese, dop Michael Ballhaus), and the second clip (51 s) was the handheld camera shot from the opening scene of “Children of Men” (dir. Alfonso Cuarón, dop Emmanuel Lubezki). After this training session, participants were given a chance to ask questions. This was followed by the actual experiment, where the subjects watched the three clips from their assigned set. In the beginning of each clip, a fixation cross for 1,000 ms was displayed. The subjects were given 40 s to fill out the assessment form after each clip, after which, the next clip was automatically started. The experiment took place in a silent editing room located at the Baltic Film and Media School of Tallinn University. The subjects watched the clips alone, with lights off, on a 27-inch iMac computer.

### 2.4. Measures and statistical modeling

Participant experience assessment relied on self-reported measures in the form of a rating task. For emotional valence and arousal, we used a 5-scale version of the Self-Assessment Manikin (SAM) (Lang, 1980; Bradley and Lang, 1994), which consists of two scales that depict five manikins (stylized human figures). For valence, their expressions range from very pleasant to very unpleasant, and for arousal from very excited to very calm. These two scales were presented visually, without numerical values, arranged from left to right. For the statistical analyses, we coded them as ranging from 1 to 5, where 5 is the most pleasant and most exciting. As for the assessment of the involvement concerning the embodiment of the camera, following Heimann et al. (2014), we asked the following three questions:

1. On a scale of 1 to 5, how much did you feel involved in the scene?
2. On a scale of 1 to 5, how much did you feel as if the camera was your own eyes?
3. On a scale of 1 to 5, how much did you feel as if you were moving with the camera? (Was not asked for the static shots)

Here, the values were accompanied by the following descriptions: “Didn't feel at all”, “Didn't feel”, “Neutral/Unsure,” “Felt”, and “Felt strongly”.

We employed a mixed-effects generalized linear regression framework to test the effect of movement on the five reported ratings (implemented using the lme4 and lmerTest packages in R; cf. Bates et al., 2015; Kuznetsova et al., 2017). While all outcome variables are technically ordinal, the linear model's assumptions are sufficiently met. In all cases, participants were modeled as a random effect, to account for repeated measurements (due to model convergence issues likely stemming from small data, we were only able to fit models with just random intercept). The movement was dichotomized as a binomial variable to simplify analysis (all moving cameras contrasted with the baseline of the static camera). We did not have any particular hypotheses concerning the relationship between mood and the outcome variables (emotions and involvement), so it was treated as a control variable (with ambiguous mood as a baseline). To assess whether movement affects the outcome variables in the respective models, we used a stepwise likelihood ratio test approach, comparing the mood model to a null (random effect only) model, and the full model (mood in interaction with movement) to the mood-only model. This, therefore, also indicates whether mood (averaging across movements) had an effect on the response, which we also consider interesting. We furthermore allow for interaction between mood and movement, assuming that effect may vary between scene types (as it indeed does, as indicated in Figure 2). The modeling differed slightly for Question 3, which concerned movement and was not asked about static scenes. Here, we followed the same stepwise procedure and included movement as a full 3-level variable (with the dolly as the baseline). In some cases, we carried out exploratory modeling on within-condition differences of interest (such as differences of the camera between a single scene), using a fixed-effects only linear regression approach that makes it convenient to report beta coefficients along with conventional *p-*values (while those data subsets do not include repeated measurements, given the nature of the sampling as described above).

## 3. Results

As discussed in the Introduction section, this study was motivated by multiple goals: to carry out experimentation on the effects of camera movements on viewer responses, but also to evaluate the suitability of experimentation for testing cinematographic theories such as embodiment (as discussed above). We, therefore, provide both statistical modeling for the movement-related hypotheses as well as exploratory results, accompanied by interpretation based on tacit cinematographic experience. Table 2 lists the results from the modeling as described in the methods section above in the form of likelihood ratio test *p*-values. In summary, after controlling for repeated measures and possible mood variation, movement in contrast to the static camera did not appear to significantly affect either valence or arousal ratings. Movement and static differ in terms of the perceived degree of involvement (Q1) and the sense of seeing the scene through one's own eyes (Q2), and the direction of the effects depends on the mood. Ignoring movement effects, we observe that valence and arousal differ between mood scenes (as somewhat expected). Given the multiple testing scenarios (five distinct questions), we also applied the Bonferroni correction (adjusting alpha to 0.01), but this ended up not affecting the interpretation.

**Table 2:** Modeling outcomes as the likelihood ratio test *p*-values from stepwise comparisons of mixed-effects regression models (mood-only against the intercept-only null model, and subsequently the full model against that).

**Model**	**Valence**	**Arousal**	**Q1, involved**	**Q2, own eyes**	**Q3, moving with the camera**
Mood-only vs. null	**< 0.001**	**< 0.001**	0.28	0.66	0.72
Mood^*^movement, vs. mood-only	0.84	0.21	**0.006**	**< 0.001**	0.99

Values below the convtentional alpha of 0.05 are highlighted in bold. ^*^ stands for interaction.

### 3.1. Valence and arousal ratings

**Figure 3:**
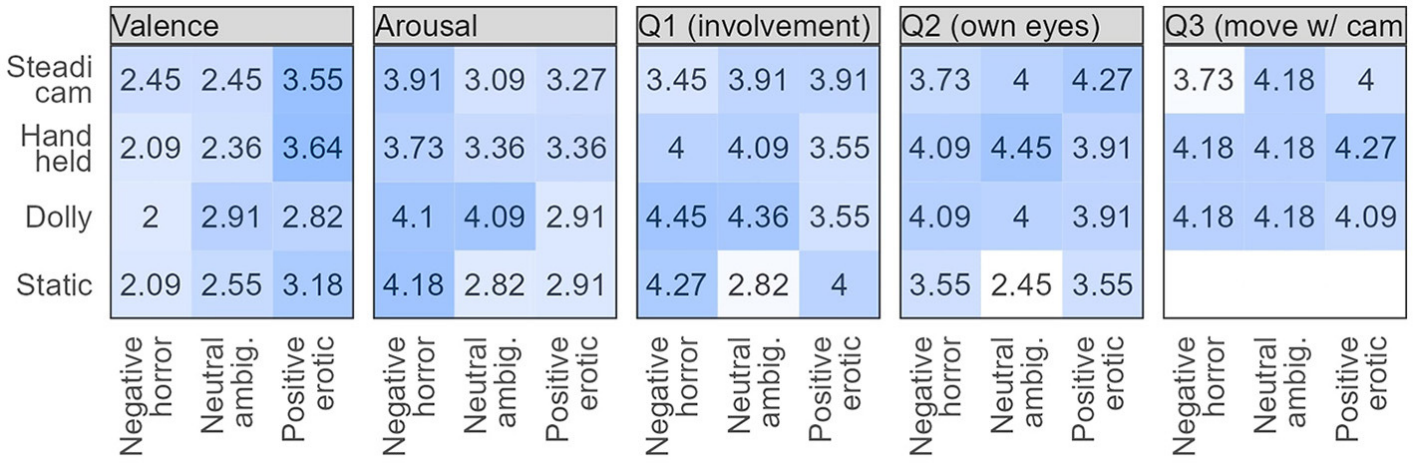
Mean rating values, ranging between 1 and 5, for combinations of camera movement and mood scene, averaged across participants. Values closer to 5 (darker background) correspond to a stronger response, e.g., the horror scene elicits stronger arousal than the erotic scene. In questions 1 and 2, moving cameras, in contrast to a static camera, lead to different immersion ratings.

Irrespective of different types of moving or static frames, the overall atmosphere generated by the set dressing appeared to be the main agent which led the viewer to perceive the scene as either pleasant or unpleasant in valence (the mood-only model in comparison to the null model: χ^2^(2) = 33.41, *p* < 0.001). The averages of all condition combinations are illustrated in Figure 3 for valence as well as other questions. In other words, whether the camera moves by any means or stays stationary did not result in the audience feeling more or less pleasant when the nature of the scene is already comprehensible (valence, controlling for mood, compared to mood-only model: χ^2^(3) = 0.84, *p* = 0.84). The only exception to this pattern seems to be the erotic encounter (positive) scene shot by the dolly movement (mean valence 2.82), which is slightly lower than other camera types (but only the difference to handheld is significant; fixed-effects only linear model with the dolly as a baseline: handheld β = 0.82, *p* = 0.03).

In general, arousal does not appear to be strongly affected by a moving camera compared to a static camera (full model compared to mood-only: χ^2^(3) = 4.56, *p* = 0.21). As can be expected, the suspenseful horror scene with blood on the floor readily elicits stronger arousal than its calmer counterparts though. In the case of an erotic encounter (pleasant) scene, dolly movement and the static frame (both averaged 2.91) would appear to lead to calmer reactions, but this difference is not statistically significant. If this difference would be replicated with a larger sample, then one explanation could be the smooth and stable frame generated by the dolly and stationary camera, which are qualities that match with pleasant onscreen events in this case.

While there was no significant difference within the horror scene, the ambiguous scene (neutral) where the onscreen clues were not evident enough for meaning-making, elicited a difference in the dolly condition (β = 1.27, *p* = 0.02, compared to static). This can be explained by the anticipation of something being revealed soon, thus adding the excitement of “diving into the unknown” (as expressed by one of the participants). In a situation where the audience is uncertain about what to expect, the mobile frame may embody active exploration of the space, whereas the static frame provides a static point of observation.

The same qualities attributed to the dolly movement and the static frame, namely smoothness and stability, can both initiate excitement and calmness simultaneously. The perception and influence of such features depend on the circumstances under which the viewers are exposed to them. For instance, the exact same dolly movement that implies a presence of an ominous entity in a horror scene can also be an invitation to join the intimacy of an erotic encounter. In cinematographic practice, different camera movements must be taken into consideration together with the settings they are executed in, including components such as music, acting, directing, and sound. As Sobchack simply puts it, “the moving becomes particularly meaningful in every film's specific context” (Sobchack, 2016, p. 86).

### 3.2. Degree of involvement

Question 1 of the involvement set of questions was stated as follows: on a scale of 1 to 5, how much did you feel involved in the scene? As indicated in Table 2, the model that includes movement describes the data better than mood only, i.e., a moving camera elicits different reactions from participants compared to static (χ^2^(3) = 12.41, *p* = 0.006). However, as illustrated in Figure 2, the effect depends on the scene. In the neutral scene, all moving cameras increase the feeling of involvement compared to static (all *p* ≤ 0.01). In other scenes, moving cameras do not differ from static. In the full model (where moving cameras are aggregated and compared to static), the interaction between mood and camera movement is a significant effect. The beta coefficients for the two interaction terms of moving camera with the non-neutral moods are both about −1.4. Through a borderline result, it further illustrates the need to consider camera movements in the context of the scene.

Question 2 asked, “On a scale of 1 to 5, how much did you feel as if the camera was your own eyes?” The moving camera effect was significant compared to the mood control (χ^2^(3) = 27.18, *p* < 0.001). Similarly to Q1, this was mostly driven by the neutral condition, with stronger immersion from moving cameras. This may be explained as an apparent correspondence between the camera movement in the space and the subject's visual perspective. This suggests that movement enhances the embodiment of the camera as a kind of extension of the viewer's eye.

Finally, question 3 was intended as explorative, to probe the differences between moving cameras, without comparison to the static condition (where this question was omitted). It asked, “On a scale of 1 to 5, how much did you feel as if you were moving with the camera?” Responses to question 3 revealed little to no difference between camera movements (movement variable with three levels, dolly as baseline; full model vs. mood only: χ^2^(6) = 3.02, *p* = 0.81). The Steadicam average in the horror scene has the lowest value, but that difference is not statistically significant.

In summary, the experimental data paints a varied picture. Camera movement might not necessarily affect valence and arousal responses in audiences, although the scene and set dressing often do, as may be expected. Movement does seem to make a difference in terms of immersion, particularly in certain types of scenes, but participants did not pick up on the individual differences between the subtly different camera techniques when asked how much they felt like they were moving with the camera. The scores were similarly high across the board. This is an interesting finding in light of the theories and tacit experience accounts discussed above and invites future research on the topic.

## 4. Discussion

The empirical focus of our study has been on understanding how the viewers experience different camera movements in dramatized scenes with different moods. Any film viewing experience can be argued to build on the anticipation of future events based on previous events in the film as well as the accumulated knowledge due to lifelong experience of films and narratives (Kauttonen et al., 2014). The knowledge related to film genre conventions (i.e., media literacy), the way how films are structured, their audiovisual design, the character's appearance, and how the plot binds it all together dictates what type of experiences the viewers expect to be engaged with.

Our findings partially support the hypothesis that a moving camera enhances viewer engagement and the feeling of being involved more than a static camera. However, emotional responses of arousal and valence were similar between moving and static camera conditions, while the content of the three scenes appeared to make a difference. The findings are in line with the neuroscientific study by Tikka et al. (2018), where it was shown that the emotionally loaded narrative content of the film scene may override the formal differences between the scene representation (audiovisual vs. written representation). We hope that this contribution will inspire experimental designs in further studies. Some limitations and shortcomings (discussed below) were noted during the process of the experiment and the data analysis based on various feedback both from participants and colleagues.

### 4.1. Filmmakers' tacit knowledge

Cinematographers tend to draw upon their inherited or tacit knowledge, the pool of information built up by filmmakers throughout the history of moviemaking. It consists of “rules of thumb that have been passed around as the 'right' or best' way to construct shots and sequences” (O'Leary, 2003, p. 199). Such knowledge could be acquired over time through practice, or simply transmitted from master to apprentice. It is not a definite set of rules, but rather an ever-evolving collective endeavor. Individual experiences also add idiosyncratic layers to this knowledge. In the short term, it can be developed through “shorter, momentary windows of revelation”, and in the longer term, it “is shaped by the experiences and collaborations a person has encountered” (Lotman, 2021, p. 34). Cinematographer Robert Richardson (ASC) explains that as his career progressed, his decisions in regard to moving the camera became “less bound to a sort of 'from the hip' sensibility” and more attentive (Pavlus, 2003, p. 41). Along the same line, his colleague in cinematography, Owen Roizman (ASC), reveals that he leaves the decision of whether to move the camera to his instincts and the camera moves only when it feels right (Calhoun, 2003).

When filmmakers pursue their “gut feeling”, they may, in fact, be applying accumulated tacit knowledge (Lotman, 2021, p. 148) from lifelong experiences “transformed into learning through a cycle of learning involving experiencing, reflecting, thinking, and acting” (Kolb and Yeganeh, 2016, p. 101). “Tacit knowledge has a personal quality, which makes it hard to formalize and communicate” yet it is “deeply rooted in action, commitment, and involvement in a specific context” (Nonaka, 1994, p. 16). Attempting to understand movies through theories alone without considering filmmakers' tacit knowledge, particularly of what “camera movements can or ought to do”, is unproductive since “filmmakers are rarely gripped by theories” (Morgan, 2016, p. 243). Or in Vorkapich's words, “what is there obtained perhaps accidentally should be sought, studied, and used consciously” (Vorkapich, 1930, p. 31). In this study, we attempted to measure and quantify what effect such intuitive decisions may have on the eventual audiences, by constructing a controlled yet relatively naturalistic experimental setting.

While a central aspect of filmmakers' tacit knowledge, camera moving techniques and their effects may be too subtle to be distinguished by non-professional viewers (Bordwell, 1977) and the relationship between camera movements and the human perceptual system went underexplored for a while (as noted by Sobchack, 1982), we see camera movements as a fruitful field of study for cognitive science and neurosciences, which in turn may also assist filmmakers in their art.

### 4.2. Limitations of the study

Our goal was to find a balance between creating natural, film-like stimuli, and retaining reasonable control over experimental variables to enable the quantification and interpretation of the results. We attempted to minimize the effect of potentially distracting variables and keep the stimuli scenes largely constant in terms of duration, pace, framing, lighting, focal length; the absence of a human agent, sound, and music). However, it can be assumed that viewers can still be affected by every component of shot design and engage with anything on the screen regardless of the intended contrast between the conditions. In addition, ordinary fiction movies do not usually lack (human) agents, sounds, or music. Therefore, while our stimuli are life-like, they might not look quite like typical movies. It is possible that in a more conventional cinema experience, the differences between camera movements could be less noticeable or vice versa.

Unlike some prior work (as discussed above), we set out to run the experiment in three scenes differentiated by mood and setting. This complicates the analysis, as the mood variable needs to be accounted for, but also allows for a richer interpretation, which we hope will inspire future research. Due to the temporal and financial limitations of a live experiment with human participants, we opted to have the same participant viewing multiple clips. While we control for these repeated measures in the statistical models, the small amount of data gathered here does not afford statistically reliable modeling of the sequence in which they watched the clips, which has the potential of affecting reactions. Future research should either seek to gather more data for more robust modeling or avoid showing participants multiple clips in succession. After the experiment procedure, some of the subjects orally expressed that irrespective of the content and the visual dynamics onscreen they felt excited simply because they did not know what they were about to see. Such excitement may have had an effect on arousal ratings, but we control for that in the statistical models via the random effects structure.

Proposing a cinematographer-oriented theory yet conducting an audience-oriented experiment might be considered contradictory. However, cinematographers devise the filmic world to manipulate viewers. In this sense, the cinematographer is the sender and the film viewer is the receiver. Whether a cinematographer succeeds in transmitting their perceptual bodily senses can only be understood by studying the receiver audience. The crux here was to design the experiment and stimulus with the cinematographer's insights. That being said, our experience of the process (including stimuli creation, experimental design, and data analysis) revealed that focusing on the creative process of the cinematographers might have been a better approach.

One of the shortcomings is related specifically to Steadicam. Steadicam Operator is a particular film profession that requires years of training to reach an adequate skill set and professional level of experience. The more skilled and experienced the operator, the more the Steadicam movement feels fluid and stable. In contrast, the floating of the horizontal line and excessive suggestion of human presence behind the device indicates an amateur operation of Steadicam. In our case, the Steadicam operator was somewhat novice; and in some Steadicam clips, the horizontal axis was not always straight, possibly resulting in a feeling of floating, which did not represent the usual fluidity of Steadicam shots.

## 5. Conclusion

In this study, we explored the views of embodied cognition on the interaction between the visual system and locomotion and sought to understand the effects of different camera movement techniques (which lead to differing camera movements on the screen) and self-reported viewer responses in terms of emotion and immersion. We explored the difference between a static and a moving camera, with three types in particular: Steadicam, dolly, and handheld, and their functions in narrative filmmaking. The insights from several professional cinematographers based on their tacit knowledge were reflected upon from the point of view of embodied simulation and embodied mind.

We highlight the utility of combining theory, professional experience-based accounts, and rigorous experimentation in controlled laboratory settings. The question remains, how exactly do the embodied experiences of the viewers and the embodiment of the tacit embodied knowledge of the cinematographers coincide in the filmmaking practice? The knowledge gained here will serve as a stepping stone for developing a more thorough understanding of embodiment, camera movement techniques, and also experimental methodologies to study them.

## Data availability statement

The datasets presented in this study can be found in online repositories. The names of the repository/repositories and accession number(s) can be found in the article/supplementary material.

## Ethics statement

Ethical review and approval was not required for the study on human participants in accordance with the local legislation and institutional requirements. The patients/participants provided their written informed consent to participate in this study.

## Author contributions

MY designed the experiment, conducted the data collection, and wrote the manuscript as the first author. PT contributed to the experiment design and writing of the manuscript. EL contributed to the experiment and stimuli designs. AK conducted the statistical analysis, wrote the relevant sections, and contributed to the writing of the manuscript. All authors contributed to the article and approved the submitted version.
